# Safety and effectiveness of oral misoprostol for induction of labour in a resource-limited setting: a dose escalation study

**DOI:** 10.1186/s12884-017-1483-5

**Published:** 2017-09-08

**Authors:** Marilyn Morris, John W. Bolnga, Ovoi Verave, Jimmy Aipit, Allanie Rero, Moses Laman

**Affiliations:** 1 Department of Obstetrics and Gynaecology, Modilon General Hospital, Madang, Papua New Guinea; 2 Department of Paediatrics, Modilon General Hospital, Madang, Papua New Guinea; 3 Papua New Guinea Institute of Medical Research, Madang, Papua New Guinea

## Abstract

**Background:**

Oral misoprostol as an induction of labour (IOL) agent is rapidly gaining popularity in resource-limited settings because it is cheap, stable at ambient temperatures, and logistically easier to administer compared to dinoprostone and oxytocin. We aim to investigate the safety and effectiveness of a regimen of oral misoprostol in Papua New Guinean women undergoing IOL.

**Methods:**

As part of a prospective dose escalation study conducted at Modilon Hospital in Papua New Guinea, women with a singleton pregnancy in cephalic presentation and an unfavourable cervix who gave written informed consent were administered oral misoprostol, commencing at 25mcg once every 2 h for 4 doses and increased to 50mcg once every 2 h for 8 doses within 24 h. The primary outcomes studied were i) the proportion of women delivering within 24 h of oral misoprostol administration, and ii) rates of maternal and perinatal severe adverse events.

**Results:**

Of 6167 labour ward screened admissions, 209 women (3%) fulfilled the study inclusion criteria and underwent IOL. Overall, 74% (155/209 [95% confidence interval 67.6–79.9]) delivered within 24 h. Most women (90%; 188/209; 95% CI [84.9–93.5]) delivered vaginally with 86% (180/209) having a good outcome for both the mother and baby. Of the 10% (21/209) who failed IOL and underwent caesarean section, a significant proportion of their babies were admitted to special-care nursery compared to babies delivered vaginally (20/21 [95%] versus 8/188 [4%]; Fisher Exact test *P* < 0.001), but their perinatal mortality rate was not significantly higher (1/21 [5%] versus 2/188 [1%]; *P* = 0.30). The only maternal death was not study related and occurred in a patient with post-partum haemorrhage, 15 h post-delivery.

**Conclusion:**

The oral misoprostol regimen for IOL described in the present study is safe, effective and logistically feasible to administer in a resource-limited setting.

## Background

Reducing maternal mortality remains a major public health challenge in many developing countries worldwide. Since the declaration of the Millennium Developmental Goals (MDG) in 2000, the implementation of MDG 5, which aims to reduce maternal mortality by three-quarters by the year 2015, has been a major undertaking in most developing countries, with significant progress in some countries but not all [1]. As recently highlighted in the Sustainable Development Goals (SDG3), maternal mortality remains 14 times higher in developing countries compared to developed countries [2]. Papua New Guinea (PNG), a developing country in the Southwest Pacific region has one of the highest maternal and perinatal mortality rates in the world [3–5]. In PNG, obstetric complications as a result of delayed presentation are common, often as a result of multiple factors including geographic remoteness, difficult logistics, limited infrastructure and a weak healthcare system. Consequently, complications such as prolonged labour are often life-threatening for women and their babies.

Induction of labour (IOL) is a critical life-saving intervention that reduces adverse outcomes. Current regimes using intravenous oxytocin and prostaglandins have been shown to be effective in inducing labour [6]. Dinoprostone, a prostaglandin E2 agent, is widely used in developed countries and approved by the Food and Drug Administration (FDA) [7], but randomised trials have failed to show that it reduces rates of caesarean sections despite its benefit as a cervical ripening agent [8, 9]. Furthermore, its high cost and instability at ambient temperatures limits its use as a cost-effective agent, particularly in resource-limited settings.

In recent years, misoprostol, a prostaglandin E1 analogue, manufactured and marketed in the United States in the 1980’s primarily to prevent peptic ulcer disease caused by the use of non-steroidal anti-inflammatory drugs [10, 11], has becoming widely used as an IOL agent. After its incidental discovery of causing uterine contractions in early pregnancy, the use of misoprostol in obstetrics has gained much interest. In April 2002, the FDA revised its original labelling for misoprostol and approved its use in pregnancy [12]. Compared to other prostaglandins, misoprostol has several potential advantages. It is stable at room temperature, inexpensive, and can be administered via several routes (oral, vaginal, sublingual and buccal).

However, currently limited data exist concerning the safety, effectiveness and feasibility of administering oral misoprostol in routine clinical practice in resource-limited settings such as PNG where the burden of obstetric and perinatal complications remain high [3]. In view of this, we developed a standard oral misoprostol protocol and assessed its safety and effectiveness as an induction agent in women attending Modilon General Hospital in Madang Province of PNG.

## Methods

### Study design and setting

This prospective dose escalation study was conducted at Modilon Hospital over a 30-month period between January 2013 and June 2015. Modilon Hospital is a Provincial Referral Hospital situated along the north coast of mainland PNG, serving a population of approximately 450,000 [13]. It provides antenatal care, family planning services and supervised delivery for the majority of the urban, peri-urban and rural populations in Madang [4, 5]. This facility also serves as a referral hospital for smaller neighbouring Provinces. The hospital delivers an average of 3000 babies per annum with approximately 3–5% of women undergoing induction of labour. In Madang Province, rates of maternal and perinatal mortality are high [3, 5], mainly due to limited accessibility and availability of health services, particularly for rural patients, compounded by difficult logistics and a weak healthcare system [14, 15].

### Study inclusion and exclusion criteria

Women delivering at the hospital with an indication for IOL such as post-date, pre-labour rupture of membranes (PLROM), pre-eclampsia, or suspected fetal compromise such as intrauterine growth retardation were considered eligible for enrolment if they fulfilled the study inclusion criteria which included i) third trimester singleton pregnancies, ii) confirmed cephalic presentations, iii) a Bishop’s score of <6, and iv) written informed consent.

Women with multiple pregnancies, preterm pre-labour rupture of membrane, previous caesarean section, malpresentation and a history of previous hypersensitivity to misoprostol or prostaglandin analogues were excluded. This study was approved by the Modilon Hospital Ethics Committee (MHEC 1502).

### Oral misoprostol study protocol

**Fig. 1:**
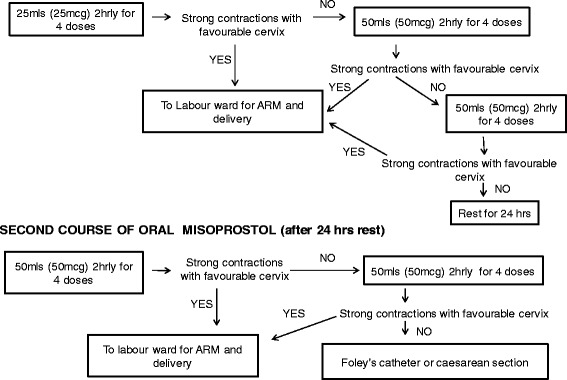
Study protocol for oral misoprostol induction of labour used in the present study

A solution of 1mcg/ml was made by dissolving 200mcg of misoprostol (200mcg per tablet, Cytotec, Piramal Healthcare, United Kingdom) in 200mls of tap water. The misoprostol solution was adequately stirred and prepared under sterile conditions, measured and given in titrated doses as per the study protocol (Fig. 1). The misoprostol solution was kept at the nurse’s station at room temperature and discarded if not completed within 24 h. Each dose, either 25mcg (25mls) or 50mcg (50mls) was given at an interval of 2 h in accordance with WHO recommendations of time-interval between each oral misoprostol dose [16].

Details of the administration of oral misoprostol are outlined in Fig. 1. Briefly, the drug was administered commencing at 25mcg once every 2 h for 4 doses and increased to 50mcg once every 2 h for 8 doses within 24 h. If there was no progression in labour, the participant was rested over the subsequent 24 h and the cycle was recommenced the following day, commencing at 50mcg once every 2 h for a total of 8 doses. If there was still no progression in labour after 8 doses of 50mcg, Foley’s catheterization was performed where indicated, or the IOL was considered failed and the participant underwent caesarean section.

### Clinical procedures

Maternal observations were taken prior to each dose with duration and frequency of contractions measured by abdominal palpation and timed with a wrist watch. Fetal monitoring was performed by listening to the fetal heart using a fetoscope and/or fetal doppler. This was also performed prior to each dose and for 30 min after an administered dose. Any changes in fetal heart rate with hypertonus (defined as a single contraction lasting more than 2 min) or tachysystole (contraction frequency of more than five within 10 min for two consecutive 10-min periods) were reported immediately for further assessment and an emergency caesarean was performed if fetal compromise was suspected [17]. Hyperstimulation was defined as tachysystole or hypertonus with non-reassuring fetal heart rate changes [17].

If the woman was assessed to have strong regular contractions, defined as 3 in 10 min with each lasting more than 30 s, [17], the next dose was withheld and she was allowed to progress to the labour ward for safe delivery and amniotomy when the Bishop score improved (score > 6). Amniotomy was not done routinely for all mothers but only when additional augmentation was required or delivery was imminent. Maternal and fetal observations were continued hourly in the labour ward and the progress of labour was assessed using the partogram as per PNG national guidelines [18]. To reduce the risk of uterine rupture, augmentation of labour with intravenous oxytocins was considered only when indicated [18], and not less than 6 h after the last misoprostol dose.

According to the study protocol, failed IOL was defined as one that did not result in vaginal delivery after completion of 2 courses of misoprostol resulting in caesarean section. This included an emergency caesarean section done at any time during the course of treatment due to suspected fetal compromise, suspected cephalopelvic disproportion, uterine hyperstimulation or suspected uterine rupture. The decision to progress to Foleys catheterization was considered only when both maternal and fetal conditions were assessed to be stable and that the membrane was intact. Failed induction with Foleys catheter was defined as not achieving vaginal delivery within 36 h of insertion [17].

### Outcomes measured

The main outcomes studied were i) the proportion of women delivering within 24 h, and ii) rates of maternal and perinatal severe adverse events such as uterine hyperstimulation, uterine rupture, maternal death and perinatal death. Other maternal outcomes measured included the duration from first dose to delivery as well as the incidence of maternal complications such as postpartum haemorrhage and retained placenta. Other fetal outcomes measured included Apgar score ≤ 7 at 1 min, at 5 min, and admission to special-care nursery.

### Data analysis

The data were analysed using STATA 11.0 analysis program (Stata Corporation, College Station, TX). Two-way comparisons of proportions were made by Fisher’s exact test. Comparison of two independent samples was by the Mann-Whitney *U* test, and comparison of multiple samples was by the Kruskal-Wallis test. A two-tailed significance level of *P* < 0.05 was used throughout.

## Results

### Patient characteristics

During the study period, a total of 6167 patients were screened, of which 209 (3%) fulfilled the study inclusion criteria and were enrolled. Their overall median age was 27 (interquartile range 24–33) years. Women who failed IOL had a higher descent on abdominal palpation at the time of admission to labour ward compared to those with a successful IOL (Fisher’s Exact test *P* = 0.03; Table 1) and though age was not significantly different between the two sub-groups, those who failed IOL were more likely to be older (median age 32 versus 27; *P* = 0.06). The 21 women who failed IOL all had a Bishop’s score of ≤5, with the majority (48%) having a score of 3. Seventeen women (8%), majority with pre-eclampsia (Table 2), had a gestational age < 37 weeks, while 100 (48%) were unbooked and did not attend at least one antenatal clinic prior to delivery. The overall median time from induction to delivery was 15 (10.5–24.4) hours. Other baseline demographic and clinical characteristics are shown in Table 1.

**Table 1:** Demographic characteristics of study participants according to delivery category. Data are numbers (percentage) or median [interquartile range]

Characteristics	Successful IOL	Failed IOL	*P*-value
Number	188	21	
Age (years)	27 [24–32]	32 [27–34]	0.06
Parity	1 [0–2]	1 [0–2]	0.63
≥4 children	28 (15)	1 (5)	0.32
Unbooked at antenatal clinic	91 (48)	8 (38)	0.49
Previous post dates	8 (4)	2 (10)	0.27
Previous still births	3 (2)	3 (14)	0.01
Previous neonatal deaths	8 (4)	0 (0)	>0.99
Previous obstetrics complications	21 (11)	5 (24)	0.15
Bishop’s Score	3 [2–4]	3 [2–3]	0.39
Descent (fifths)	4 [4–5]	5 [4–5]	0.03
Ultrasound confirmation of fetal lie	97 (52)	14 (67)	0.25
Systolic blood pressure	120 [100–122]	120 [118–130]	0.12
Diastolic blood pressure	80 [70–90]	80 [80–90]	0.17

**Table 2:** Comparison of maternal and perinatal outcomes according to indications for induction of labour. Data are numbers (percentage), median [inter-quartile range]

Maternal and perinatal outcomes	Post-dates	PLROM	Pre-eclampsia	Fetal compromise^a^	Fetal death in-utero	Others	*P*-values
Number	117	45	28	10	7	2	
Preterm (<37 weeks)	0 (0)	5 (11)	11 (39)	0 (0)	0 (0)	1(50)	<0.001
Failed induction	11 (9)	5 (11)	2 (7)	3 (30)	0 (0)	0 (0)	0.33
Delivered within 24 h	76 (65)	35 (78)	9 (32)	6 (60)	2 (29)	1 (50)	0.26
Duration of labour (hours)	9.0 [5.8–12.3]	6.4 [4.0–10.3]	9.3 [5.9–12.1]	9.8 [6.9–17.0]	10.0 [5.7–16.3]	14 [13.4–14.6]	0.07
Haemoglobin (g/dL)	10.1 [9.3–12.2]	10.5 [8.4–12.0]	10.3 [9.5–12.6]	10.0 [8.2–12.2]	8.0 [7.0–8.9]	7.4 [1.7–13.0]	0.15
Positive VDRL test	3 (3)	3 (7)	0 (0)	0 (0)	0 (0)	0 (0)	0.32
Maternal complications							
*Postpartum haemorrhage*	7 (6)	3 (7)	4 (14)	1 (10)	1 (14)	0 (0)	0.71
*Retained placenta*	0 (0)	2 (4)	0 (0)	0 (0)	2 (29)	0 (0)	<0.001
*Uterine rupture/hyperstimulation*	0 (0)	0 (0)	0 (0)	0 (0)	0 (0)	0 (0)	–
*Maternal death*	0 (0)	0 (0)	1 (4)	0 (0)	0 (0)	0 (0)	0.26
Perinatal outcomes							
*Apgar score ≤ 7 at 1 min*	13 (11)	7 (16)	7 (25)	2 (20)	NA	0 (0)	0.04
*Apgar score ≤ 7 at 5 min*	3 (3)	2 (4)	5 (18)	0 (0)	NA	0 (0)	<0.001
*Baby admitted to SCN*	12 (10)	10 (22)	4 (14)	2 (20)	NA	0 (0)	0.51
*Perinatal death*	0 (0)	2 (4)	1 (4)	0 (0)	NA	0 (0)	0.33

^a^ Includes severe intra-uterine growth restriction, *NA* not applicable, *PLROM* Pre-labour rupture of membrane

### Indications for IOL

The majority of women underwent IOL for post-dates (56%; 117/209). Forty-five (22%) had pre-labour rupture of membranes (PLROM), 28 (13%) had pre-eclampsia and 10 (5%) had suspected fetal compromise including intrauterine growth restriction (Table 2). Seven women (3%) had fetal death in-utero, while other indications such as gestational diabetes and severe anaemia as a complication of partial abruption accounted for the remaining 2 patients (1%).

### Maternal outcomes

**Fig. 2:**
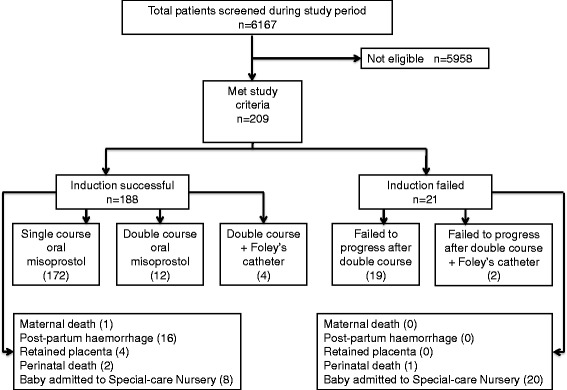
Flowchart of study participants outlining outcome for each delivery category

Ninety percent of women (188/209) had a successful vaginal delivery compared to 10% (21/209) who failed IOL and underwent caesarean section. Of those with a successful vaginal delivery, 74% delivered within 24 h with no significant difference between the various diagnostic categories (Table 2). The majority of deliveries (86%; 180/209) resulted in a good outcome for both mothers and babies (Table 2). Of the 6 women who had Foley’s catheterization after two courses of misoprostol, four delivered vaginally while 2 failed to progress and underwent emergency caesarean section due to suspected fetal compromise (Fig. 2). Of those receiving misoprostol without Foley’s catheterization, 91% (184/203) delivered vaginally (Fig. 2). The primary reasons for operative delivery were failed IOL and/or prolonged labour (62%; 13/21) and suspected fetal compromise (38%; 8/21).

**Fig. 3:**
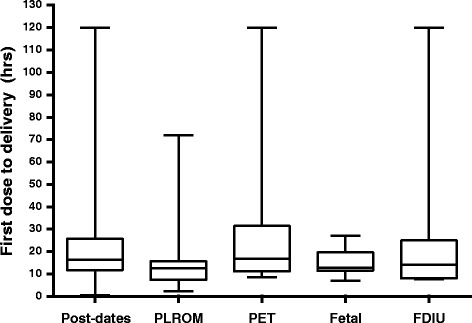
Box plot showing median, interquartile range, minimum and maximum duration between commencements of induction to delivery, according to diagnostic indications for labour induction. Footnote: PLROM = pre-labour rupture of membrane, PET = Pre-eclampsia, Fetal = Fetal compromise, FDIU = Fetal death in-utero. The Others group containing only 2 patients is not included

Majority of women delivered after the first course of oral misoprostol compared to those who delivered after the second course (91% [172/188] versus 9% [16/188]; *P* < 0.001). Categorically, the shortest median time from administration of the first dose to delivery was documented in women induced for PLROM (Fig. 3; Kruskal Wallis test *P* = 0.03). Thirty-eight women (18%) required augmentation with intravenous oxytocin for poor progress of labour owing to ineffective contractions.

### Maternal complications

Maternal complications were documented in 21 women (10%). Four had retained placenta requiring manual removal and half of these women had fetal death in-utero (Table 2). Sixteen women had postpartum haemorrhage but this was not significantly associated with any diagnostic category (Table 2) or the type of delivery (caesarean delivery [0/21] versus vaginal delivery [16/188]; *P* = 0.38; Fig. 2). There was a maternal death in a 39 weeks primigravida who had pre-eclampsia and fetal death in utero. She delivered a macerated stillbirth baby weighing 2.5 kg and suffered a secondary postpartum haemorrhage of more than 2 l. Despite emergency fluid and blood resuscitation, she went into cardio-pulmonary arrest and died 15 h post-delivery. There were no cases of uterine rupture or hyperstimulation.

### Perinatal outcomes

The majority of babies (99%; 206/209) survived with a good outcome on discharge. Overall, a significant number of babies admitted to special-care nursery were delivered by caesarean section relative to the successful IOL group (20/21 [95%] versus 8/188 [4%]; *P* < 0.001), but there was no significant difference in perinatal mortality between the two groups (Successful IOL: 2/188 [1%] versus Failed IOL: 1/21 [5%]; *P* = 0.30). Babies born to mothers with pre-eclampsia had a higher rate of abnormal Apgar scores at both 1 min and 5 min after delivery compared to other groups (Table 2). In the successful IOL group, the 8 admissions to special-care nursery (Fig. 2) were due to low birth weight (4), neonatal sepsis (3) and congenital malformations (1). Most of the 20 babies delivered by caesarean section and admitted to nursery were admitted for routine observations (15), followed by meconium aspiration syndrome (2), neonatal sepsis (2) and severe congenital abnormalities (1). Considering admissions to special-care nursery, there was no significant difference between neonates born preterm (gestational age < 37 weeks) compared to those born at term (3/17 [18%] versus 25/192 [13%]; *P* = 0.71).

The caesarean delivered baby with severe congenital abnormalities died. In addition to her severe malformations, she was preterm. Her mother underwent caesarean section at 32 weeks because of her failed IOL and worsening severe pre-eclampsia. The remaining two perinatal deaths occurred in babies of mothers who had a successful IOL (Table 2). One had multiple congenital abnormalities while the other was a preterm, low birth weight baby.

## Discussion

The present prospective observational study shows that almost three-quarters of Papua New Guinean women undergoing IOL with oral misoprostol commencing at 25mcg once every 2 h for 4 doses and escalating to 50mcg once every 2 h for 8 doses safely delivered within 24 h. Furthermore, amongst the 209 induced women, 90% delivered vaginally and 86% had a successful maternal and perinatal outcome without complications. The only maternal death in this study was unrelated to study procedures, and there were no cases of uterine rupture or hyperstimulation. Retained placenta was commonest in women with fetal death in-utero and the rate of post-partum haemorrhage was not significantly different between diagnostic or delivery categories. Remarkably, the rate of perinatal mortality in patients undergoing oral misoprostol IOL in the present study was exceptionally low (Table 2) compared to our previously reported high rates of perinatal mortality documented as part of routine clinical practice [3]. This suggests that IOL using the regimen described in the present study is safe, effective, reduces adverse outcomes and can be easily administered in resource-limited settings, although further studies utilizing a randomised control design will be needed to confirm these findings.

The success of this regimen may be attributed to the low-dose of oral misoprostol commencing at 25mcg per dose second hourly for the first 8 h before escalating the dose to 50mcg if there was no complications and/or progression in labour. Our findings are supported by a systematic review which found low-dose oral misoprostol (≤25mcg) to be as effective and safe as vaginal dinoprostone with significantly fewer women requiring caesarean delivery [19]. Secondly, it is evident that the oral route of misoprostol administration is associated with a reduced risk of severe adverse events such as uterine hyperstimulation and rupture compared to the vaginal route [20].

Induction regimes using vaginal misoprostol have raised concerns of hyperstimulation and fetal heart rate changes due to the longer half-life and direct effect of misoprostol on the cervix [19, 21]. In addition to the route of administration and dosage, it is also apparent that duration and the time interval between doses plays an important role in the efficacy and safety of oral misoprostol in labour induction [22–24]. However, there is still a high rate of heterogenicity between studies comparing the vaginal and oral routes of misoprostol administration [25], despite many such studies being previously conducted and scrutinised in systemic reviews [19, 25]. On the other hand, there is a lack of data and there is still a need for intervention trials to compare various oral misoprostol dose regimens, with particular attention on dosage, time interval between doses and duration of treatment.

Oral misoprostol is a relatively new IOL method in many developing countries including PNG where standardised national protocols are yet to be established. Furthermore, authoritative recommendations to perform IOL which often results in 20–30% of induced deliveries in developed countries [19, 24, 26, 27] are often not practical in resource-limited settings, owing to multiple factors. Pre-hospital factors such as geographic remoteness, logistics and infrastructure limitations as well as poverty, illiteracy and cultural perspectives can significantly delay presentation of patients to hospital [15]. Additionally, the neglected healthcare system in most resource-limited settings and in-hospital factors such as poor turnaround time for emergency caesarean sections due to limited personnel, laboratory and blood bank services are common compounding factors [28]. This emphasizes the importance of IOL is an important tool, as well as the need for safer and practical IOL methods to be established in resource-limited settings.

The present study had a number of limitations. First, the study would have been stronger had there been a control group. However, the main aim of the study was to establish the safety and effectiveness of this regimen in routine clinical practice using an observational research design before we could progress to comparing this regimen with other regimens. Second, the use of oxytocin in augmenting labour in 38 women in the present study may have been a confounding factor. However, this clinical decision was deemed necessary in order to ensure a safe labour outcome. Thirdly, it is possible that under research conditions, the patients undergoing IOL received a higher care than usual resulting in the low maternal and perinatal adverse outcomes observed in the study. Nevertheless, this study was conducted by hospital-based healthcare providers as part of routine clinical practice and highlights that adverse maternal and perinatal outcomes can be reduced if IOL is carefully implemented in resource-limited settings where the burden of adverse outcomes remain high [3]. Finally, the presence of a paediatrician during caesarean section may have influenced rates of admission to special care nursery resulting in a higher number of babies being admitted for observations.

## Conclusion

Our findings suggest that the current oral misoprostol regimen described in the present study is safe, effective, reduces maternal and perinatal mortality, and can be easily implemented in resource-limited settings. In addition to being cheap and stable at ambient temperatures compared to dinoprostone and oxytocin [19], the simplicity and popularity of oral misoprostol is likely to improve IOL rates in developing countries, which will in turn reduce the unacceptably high maternal and perinatal mortality rates in these settings [1, 3]. Further intervention trials comparing the current regimen to other oral misoprostol regimens will be required to ensure appropriate safety and efficacy is maintained without increasing the risk of misoprostol-induced adverse events.

## Abbreviations

ARM: Artificial Rupture of Membranes
FDA: Food and Drug Administration
FDIU: Fetal Death In Utero
IOL: Induction Of Labour
MDG: Millennium Development Goal
MHEC: Modilon Hospital Ethics Committee
PET: Pre-eclampsia Toxemia
PLROM: Pre-labour Rupture of Membranes
PNG: Papua New Guinea
SCN: Special Care Nursery
VDRL: Venereal Disease Research Laboratory
